# West Syndrome Is an Exceptional Presentation of Pyridoxine- and Pyridoxal Phosphate-Dependent Epilepsy: Data From a French Cohort and Review of the Literature

**DOI:** 10.3389/fped.2021.621200

**Published:** 2021-03-05

**Authors:** Marc Gibaud, Magalie Barth, Jérémie Lefranc, Karine Mention, Nathalie Villeneuve, Manuel Schiff, Hélène Maurey, Marie-Anne Barthez, Isabelle Caubel, Mondher Chouchane, Diane Doummar, Manoëlle Kossorotoff, Marie-Dominique Lamblin, Agathe Roubertie, Rima Nabbout, Patrick Van Bogaert

**Affiliations:** ^1^Service de neuropédiatrie, CHU d'Angers, Angers, France; ^2^Service de génétique médicale, CHU d'Angers, Angers, France; ^3^Service de pédiatrie, CHU de Brest, Brest, France; ^4^Centre de référence des Maladies Héréditaires du métabolisme, Hôpital Jeanne de Flandre CHRU Lille, Lille, France; ^5^Service de neuropédiatrie, Hôpital de la Timone, APHM Marseille, Marseille, France; ^6^Centre de référence maladies héréditaires du métabolisme Hôpital Robert Debré, APHP Paris, Paris, France; ^7^Service de neuropédiatrie Hôpital Kremlin-Bicêtre APHP Paris, Paris, France; ^8^Service de neuropédiatrie, CHU de Tours, Tours, France; ^9^Service de pédiatrie, CH Lorient, Lorient, France; ^10^Service de pédiatrie, CHU de Dijon, Dijon, France; ^11^Service de neuropédiatrie, Hôpital d'Enfants Armand-Trousseau APHP Paris, Paris, France; ^12^Service de neuropédiatrie et maladies métaboliques, Hôpital Necker-Enfants Malades APHP Paris, Paris, France; ^13^Service de physiologie et explorations fonctionnelles, Hôpital Jeanne de Flandre CHRU Lille, Lille, France; ^14^Service de neuropédiatrie, CHU de Montpellier, Montpellier, France; ^15^Laboratoire Angevin de Recherche en Ingénierie des Systèmes (LARIS), Université d'Angers, Angers, France

**Keywords:** epilepsy, West syndrome, infantile, spasms, pyridoxine, pyridoxal phosphate, antiquitine, B6

## Abstract

**Objective:** To characterize the electro-clinical presentation of patients with pyridoxine-dependent epilepsy (PDE) and pyridoxal phosphate (PLP)-dependent epilepsy in order to determine whether some of them could be diagnosed as *de novo* West syndrome, i. e., West syndrome that starts after the age of 2 months without other types of seizures (focal seizures for instance) before the onset of epileptic spasms.

**Methods:** We analyzed data from an unpublished cohort of 28 genetically confirmed cases of PDE with antiquitine (ATQ) deficiency and performed a review of the literature looking for description of West syndrome in patients with either PDE with ATQ deficiency or PLP-dependent epilepsy with Pyridox(am)ine phosphate oxidase (PNPO) deficiency.

**Results:** Of the 28 cases from the ATQ deficiency French cohort, 5 had spasms. In four cases, spasms were associated with other types of seizures (myoclonus, focal seizures). In the last case, seizures started on the day of birth. None of these cases corresponded to *de novo* West syndrome. The review of the literature found only one case of PNPO deficiency presenting as *de novo* West syndrome and no case of ATQ deficiency.

**Significance:** The presentation of PDE- and PLP-dependent epilepsy as *de novo* West syndrome is so exceptional that it probably does not justify a systematic trial of pyridoxine or PLP. We propose considering a therapeutic trial with these vitamins in West syndrome if spasms are associated with other seizure types or start before the age of 2 months.

## Introduction

West syndrome is an epilepsy syndrome arising between 2 months and 1 year, with an incidence estimated at 2–3/10,000 of live birth (1). Epileptic spasms are the hallmark of West syndrome. These epileptic seizures are characterized by contractions of the axial musculature of variable intensity, ranging from brief head nodding to massive abduction and flexion of the upper limbs, and occurring in clusters. A very abnormal interictal EEG pattern called hypsarrhythmia, i.e., high voltage, asynchronous, slow activity mixed with multifocal spikes and sharp waves, and arrest of psychomotor development complete the classical triad of West syndrome (2). Developmental arrest may be absent at the onset of spasms and is therefore not a mandatory feature to pose a diagnosis of West syndrome. In some patients, seizures occur before the age of 2 months, showing either a semiology of spasms or a different semiology (focal seizure, myoclonic seizure, and tonic seizures). When associated with a suppression-burst (SB) pattern on EEG, these early seizures are part of early epileptic encephalopathy with SB, which is a different epileptic syndrome than West syndrome but may evolve to West syndrome with age. Some patients may also start an epileptic disorder after the age of 2 months with other types of seizures (focal seizures for instance) before the onset of infantile spasms. In the other cases (*de novo* cases), epileptic spasms are the first epileptic manifestations of West syndrome.

Etiologies of West syndrome are variable, including structural, genetic, metabolic, and unknown etiologies. The structural group includes patients with malformations of cerebral cortical development, including tuberous sclerosis complex, and those with other types of prenatal, perinatal, or postnatal lesions of ischemic, infectious, traumatic, or even tumoral origin. The genetic etiologies include chromosomal aberrations and rearrangements, and mutations of various genes (CDKL5, ARX, etc.) that are usually associated with developmental delay prior to the onset of spasms. The metabolic etiologies were recently reviewed by Salar et al. (3). The authors conclude that West syndrome has a metabolic cause in about 3–5% of the cases in developed countries, but this proportion is higher in countries with high level of consanguinity (4). According to this paper, phenylcetonuria and related disorders are the most prevalent causes, and other metabolic causes include disorders of glucose metabolism and transport, Menkes disease, mitochondrial disorders, organic aciduria, biotinidase deficiency, congenital disorders of glycosylation, and vitamin dependencies: pyridoxine (vitamin B6) and pyridoxal phosphate (PLP) dependencies.

Pyridoxine and PLP dependencies are different diseases resulting in a deficit in PLP, which is the biologically active form of pyridoxine. It is a very important vitamin for brain functioning, as it is the cofactor for many enzymatic reactions, some of them being involved in epileptogenesis through GABA and glutamate metabolism (5). Pyridoxine-dependent epilepsy (PDE) is an epileptic syndrome with heterogeneous genetic background. Most cases are related to homozygous mutation of the ALDH7A1 gene encoding for antiquitine (ATQ), a key enzyme in the metabolism of lysine (5). This disease is not exceptional, with an incidence estimated at about 1:64,000 live births, and represents the most frequent form of PDE (6). In this disease as well as in hyperprolinemia type 2, metabolites such as pipecolic acid, alpha-aminoadipic semialdehyde (AASA), and Δ^1^-piperideine-6-carboxylate accumulate. The accumulation of these compounds will result in inactivation of PLP and possibly in neurotoxicity. Other cases of PDE are related to mutations of proline synthetase co-transcribed homolog (PROSC), a protein renamed as PLP-binding protein (PLPBP) and involved in the homeostasic regulation of PLP (7). PDE can also be related to impairment of PLP import into the brain, such as in hypophosphatasia and glycosylphosphatidylinositol (GPI) anchor synthesis defects (5). Finally, PDE has been described in molybdenum cofactor deficiency (8). PLP-dependent epilepsy is rarer than PDE and is related to Pyridox(am)ine phosphate oxidase (PNPO) deficiency, i.e., the enzyme that catalyzes the reaction from Pyridoxine-phosphate or Pyridoxamine-phosphate to PLP. Patients typically respond to PLP but not to pyridoxine, though partial response to pyridoxine has been described and some patients with PNPO deficiency for unknown reasons only respond to pyridoxine (9–11). In PDE as well as in PLP-dependent epilepsy, seizures typically start in the neonatal period and patients show an association of different types of seizures that may be either generalized (spasms, myoclonic, or tonic seizures) or focal (12, 13). They may lead to drug-resistant status epilepticus. The SB pattern or a combination of continuous and discontinuous patterns was considered as suggestive of B6 dependency (14). However, SB was found in only 21% of the patients in a more recent and larger series (15). Moreover, in that series, seizures had onset after the age of 1 month in 11% of the cases. PDE related to ATQ deficiency may be screened in searching for elevated AASA in urines, the test being highly sensitive and specific (15). However, measurement of AASA in urine is only available in specialized labs and, due to its instability, requires thorough cold chain handling during transport and storage. Pipecolic acid measurement is more readily available but less specific for ATQ deficiency. As these metabolic biomarkers of PDE are not available for rapid diagnosis, trials with pyridoxine (30 mg/kg/day over 1–3 days), followed by add-on of folinic acid (3–5 mg/kg/day) if ineffective, and then switch to PLP (30–60 mg/kg/day over 3 days) if ineffective, are recommended when PDE is suspected (5).

Guidelines for the treatment of West syndrome include hormonal therapy (ACTH, hydrocortisone, or prednisolone) and vigabatrin as first-line treatments. Recently, the association of hormonal therapy and vigabatrin was shown as more efficient than hormonal therapy alone in patients aged from 2 to 14 months of age with West syndrome of recent onset (16).

Early response is a key point for the treatment of West syndrome, especially in patients of unknown etiology and normal psychomotor development prior to the onset of spasms. Indeed, early effective treatment with ACTH was associated with normal or only slightly impaired cognitive outcome in 70–100% in two reported series (17, 18). It is now well admitted that longer lead time to treatment is associated with poorer outcome (19, 20). Rapid recognition of vitamin-dependant epilepsy is also a key point for long-term outcome as early treatment with pyridoxine or PLP may lead to better psychomotor development (10, 21). Consensus guidelines for the management of PDE related to ATQ deficiency have recently been published and include the implementation of lysine restricted diet and arginine supplementation in order to improve the cognitive outcome (22). However, a trial with pyridoxine or PLP is only rarely proposed in patients with West syndrome of unknown etiology (23).

The purpose of this study is to characterize the electro-clinical features of patients with PDE and PLP-dependent epilepsy in order to determine whether some of them could be diagnosed as West syndrome, either *de novo* or following another type of epilepsy. This should prompt to propose a trial with pyridoxine or PLP in a subgroup of patients with West syndrome before starting a more classical treatment associating hormonal therapy and vigabatrin. To achieve this goal, electro-clinical data from an unpublished cohort of PDE related to ATQ deficiency from France were analyzed, and the current literature was reviewed.

## Materials and Methods

### French Cohort of Patients With ATQ Deficiency

A cohort of patients with PDE due to ATQ deficiency was recruited through an announcement diffused to the physicians participating to the Reference Center for Rares Epilepsies, based in Hôpital Necker-Enfants Malades (Paris), which constitutes a network covering almost all the country. Electro-clinical data were collected by the same physician (M.G.) from the patients' hospital medical files. Data collected focused on familial history of epilepsy, course of pregnancy, delivery, clinical signs preceding seizures, age at onset of seizures, type of first seizures, initial treatment, pyridoxine sensitivity, AED response, alternative treatments (folinic acid, lysine-restricted diet), clinical condition in the first months, seizure control, psychomotor development, EEG before and after pyridoxine administration, cerebral imaging, school performance, metabolic biomarkers, and genetics. It should be noted that there is no agreement between authors on the semiology features that allow distinction between epileptic spasms and tonic seizures in newborns. In describing the syndrome that bears his name, Ohtahara speaks about “tonic spams with or without clustering” (24). In this study, we decided to speak about epileptic spasms for seizures that occurred in clusters. Isolated events were called tonic or myoclonic seizures according to the duration of seizures and the ictal pattern on EEG if available.

### Bibliographic Analysis

We investigated in the literature the presence of cases of vitamins dependent epilepsy, which presented epileptic spasms in the course of the disease. This search was done under PubMed for English language using the following associations of keywords:

Antiquitine + (infantile spasm or west syndrome)

ALDH7A1 + (infantile spasm or west syndrome)

PNPO + (infantile spasm or west syndrome)

Pyridoxine + (infantile spasm or west syndrome)

PLPBP + (infantile spasm or west syndrome): 0

MOSC + (infantile spasm or west syndrome): 0

Hyperprolinemia + (infantile spasm or west syndrome): 0

Hypophosphatasia + (infantile spasm or west syndrome): 3

GPI anchor + (infantile spasm or west syndrome): 8

Molybdenum cofactor + (infantile spasm or west syndrome): 4.

We considered here West syndrome when there was an association of clusters of spasms arising or persisting after the age of 2 months and an EEG pattern of hypsarrhythmia. In patients without genetic confirmation of either ATQ or PNPO deficiency, we considered that the diagnosis of PDE or PLP-dependent epilepsy was likely only in patients with epilepsy that responded to one of these two vitamins and either recurred after vitamin withdrawal or presented biological markers of these diseases. We did not include patients described only in abstracts or those with limited clinical information.

## Results

###  Cohort of Patients With ATQ Deficiency

We collected a previously non-reported cohort of 28 patients with PDE due to ATQ deficiency with genetic mutations. Clinical characteristics are summarized in Table 1.

**Table 1:** Clinical description of the cases, and genetic mutations of ALDH7A1 gene.

**Case (number)**	**Age at last neurological assessment (years)**	**Age at onset of epilepsy**	**Age of pyridoxine introduction**	**Seizures types**	**Motor development/Language development**	**Genetics**
1	15	8th day	7 weeks old	Focal and myoclonic jerks	2/2	c.750G>A c.818A>T
2	12	3rd day	1 month old and 3 years	Hemicorporal right or left, generalized	2/2	c.787+5G>A c.1463A>G
3	3	11th day	11th day	Focal and myoclonic seizure	1/1	p.Glu427Gln ; 2nd mutation not found
4	10	1st day	1st day	Spasms	1/1	c. 1195 G>C homozygous
5	3,5	1st day	1st day	Focal and generalized	2/2	c. 1195G>C (p.Glu399Gln) homozygous
6	1,5	3 months old	Not administered	Focal: eyes and limbs movements		c.1459_1471dup ; c.1382G>A
7	3	2nd day	2nd day	Focal: eyes and limbs movements	2/	c.1459_1471dup ; c.1382G>A
8	29		1st day	Generalized	1/1	c.491C>T ; c.1429G>C
9	6	4 months old	5 months old	Focal, hemicorporal right, generalized, spasms	3/3	c.612-1G>T ; c.690-1095_71delinsG
10	5	1st day	3rd day	Spasms and myoclonic jerks	2/2	c.690-1095_716delinsG : r.690_787del ; c.863delC : p.(Pro288HisfsX32)
11	5	1st day	3 months old	hemicorporal right, left, generalized, spasms	1/2	c.1251delinsAAA : p.Phe417LeufsX8 homozygote
12	1	3 months	3 months	Generalized and focal	2/	c.1364T>C (p.leu455pro) homozygous
13	8	7th day	7th day	generalized	2/3	c.163-1G>C homozygous
14	1	3 months old	5 months old	Hemicorporal right or left	2/	c.1279 G>C (p.glu427Gln) homozygous
15	9	11th day	15th day	Generalized	2/3	c.818A>T : p .Asn273Ile c.1429G>C : p.Gly477Arg
16	12	1st day	12th day	Myoclonic jerks of face and limbs	2/2	c.811G>A (p.Gly271Arg) homozygous
17	5	1st day	1st day	Generalized	1/2	m c.1328_1331+9delinsGTTGGG homozygous
18	6	1st day	2 months old	Hemicorporal, generalized and erratic myoclonic jerk	1/1	c.505C>T: p.Pro169Ser ; deletion of exon 6
19	12	1st day	5th day	Hemicorporal, generalized	1/1	c.434-1G>C ; c.331delA : p.(Ile111SerfsX2)
20	5	1st day	1st day	Generalized	2/	c.434-1G>C ; c.331delA : p.(Ile111SerfsX2)
21	5	11 months old	12 month old	Focal, generalized, atonic	2/3	c.612-502G>C ; r.612-541_506
22	3	12 months old	2 month old	No seizure	1/	c.612-502G>C ; r.612-541_506
23	28	1st day	4th day	Unknown	2/2	c 750G>A ; c748-787 del
24	0	1st day	Not administered	Generalized		c.1195G>C homozygous
25	0	3rd day	Not administered	Myoclonic jerks		c.1195G>C homozygous
26	1	1st day	7th day	Generalized	1/1	2 mutations
27	1.5	22nd day	23rd day	Generalized and tonic	1/	c.612-502G>C ; c.690-1095_716delinsG
28	0.3	1st day	2nd day	Focal, tonic, myoclonic jerks and spasms	1/	c312+1G>A ; c. 1279G>C

*Motor development and language development. 1: normal, 2: moderate impairment, 3: severe impairment*.

All but one patient presented epileptic seizures, a patient diagnosed on the presence of familial history who received pyridoxine from the first day of life. The age at onset of seizures was within the 24 h after birth in 14/28 (50%), and in the first 2 months in 21/28 (75%). Pyridoxine was never started in three children, who died.

The children presented different types of seizures: focal seizures in 14 patients, myoclonic seizures in 7, generalized tonic–clonic seizures in 16, and epileptic spasms in 5. Fifteen children presented more than one type of seizure.

Epileptic spasms were associated with other types of seizures in 4/5 and started between 1 and 16 weeks of life. Only one patient showed spasms only (case # 4). In this patient, the spasms started on the first day of life, and the EEG showed a SB pattern. In the six patients with onset of seizures after the age of 2 months, only one had epileptic spasms. This patient (case # 9) started her epilepsy at the age of 4 months with focal seizures, and then presented epileptic spasms. Her psychomotor development was mildly impaired from birth, with delayed ocular fixation. Interictal EEG performed at the onset of epileptic spasms showed an atypical hypsarrhythmic pattern.

Neurological development was very often impaired. At an average age at last assessment of 7.3 years (standard deviation: 5.4), only 8 children had normal development, 12 had moderate impairment, 4 had severe impairment, and 3 had died. Data were missing in one patient.

To sum up, none of the patients from this large cohort of PDE were diagnosed as *de novo* West syndrome.

### Review of the Literature

The different combinations of searches made it possible to find 37 articles. Ten of them reported relevant cases.

In the North American cohort of PDE published by Bennett et al. (25), four patients presented with epileptic spasms. Two of them were published as K30031 and two by Battaglioli et al. (26). They are two siblings who both had epileptic spasms at 6 months of age. Results of EEG at diagnosis of epileptic spams are not provided. The boy did not develop severe epilepsy later, and his EEG was normal at age 4. Pyridoxine had a beneficial effect on language and cognition at age 8 years. His sister was treated with pyridoxine at the age of 6 months, allowing a resolution of spasms in 3 days. There was no mention of stopping pyridoxine supplementation to assert PDE. Neither mutation nor deletion in the ALDH7A1 gene was found and the level of pipecolic acid was normal in the second child. The third case (K3009) was published by Bennett et al. (27). This girl experienced epileptic spasms at the age of 3 months, resistant to ACTH and vigabatrin, but with a sustained response to pyridoxine. The search for mutation or deletion in the ALDH7A1 gene was negative and the blood level of pipecolic acid was normal (28). The fourth case is a boy having started spasms with hypsarrhythmia at 5 months that were drug-resistant but rapidly responded to pyridoxine. No mutation or deletion in the ALDH7A1 gene was found, and the level of pipecolic acid was normal (28). To sum up, these four patients presented as West syndrome that responded to pyridoxine, but genetic investigations were negative and biological markers for ATQ deficiency were absent. Therefore, we cannot conclude that these four patients had PDE presenting as West syndrome, even if PDE related to another gene, that ALDH7A1 gene remains a possibility.

The article published by Mills et al. (29) reports a child (patient 7, see additional data of this article) who started his epilepsy at 5 months of life with epileptic spasms, without other types of seizures and hypsarrhythmia on EEG. Her epilepsy was rapidly controlled by vigabatrin and PLP. When PLP was removed, the seizures recurred. Evolution was favorable for epilepsy and development with PLP monotherapy. The molecular diagnosis of PNPO deficiency was confirmed (homozygous R116Q mutation). So, the clinical presentation for this patient is *de novo* West syndrome.

In a recent article published by Salar et al. (3), the authors list the metabolic causes of West syndrome. It is mentioned in this article that the deficit in ATQ can be a cause, citing Liu et al. (30) who reported a patient with clinical response to pyridoxine but without PDE as seizures did not recur when pyridoxine was stopped.

The study published in 2018 by Xue et al. (31) describes characteristics of 744 patients with epileptic spasms. Eleven of them had a response to pyridoxine alone or had hypsarrhythmic EEG, and spasms were not preceded by other types of seizures. However, no mutation was found for the ALDH7A1 or PNPO genes, and other genes of PDE were not researched.

In a genetic study of epileptic spasms (32), there was 1 case who presented a mutation of PNPO. His epilepsy began on the first day of life, with myoclonus and focal seizures, and SB pattern on EEG. A trial of IV pyridoxine followed by oral pyridoxine was unsuccessful. PLP was not tried. He developed flexion spasms at 3.5 months of age, followed by recurrent status epilepticus. The spasms were drug-resistant. So, this patient had early epileptic encephalopathy with SB that evolved to West syndrome.

Finally, a case of West syndrome related to hypophosphatasia responding remarkably to PLP after failure of many anti-epileptic drugs was reported (33). This patient had intractable tonic seizures and a SB pattern from day 2, and epileptic spasms associated with hypsarrhythmia occurred at the age of 2 months. So, this patient did not present as *de novo* West syndrome, and had in addition clinical and radiological features of skeletal dysplasia.

## Discussion

We report here a new cohort of 28 patients with PDE and ALDH7A1 mutation. This is one of the larger series reported so far. The analysis of this French cohort did not identify any cases presenting as typical *de novo* West syndrome, i.e., onset of epilepsy with epileptic spasms after the age of 2 months and hypsarrhythmia on EEG. Indeed, the five patients who presented epileptic spasms had either early neonatal epilepsy, or other types of seizures before the onset of spasms. In addition, none of these five patients did show a typical hypsarrhythmia on EEG. The review of the literature identified a single patient with PLP-dependent epilepsy due to PNPO deficiency who presented as a typical *de novo* West syndrome (29). To our knowledge, this is the only published case, and we did not find any case of PDE presenting as *de novo* West syndrome.

There is abundant but discordant literature on the use of pyridoxine and PLP in the treatment of West syndrome whatever the underlying etiology, suggesting a non-specific anti-epileptic effect. Pyridoxine and PSP were considered as very important treatment options in a literature of the 80s−90s, mainly issued from Japan (34, 35). More recent studies are less enthusiastic. Debus et al. (36) did not find any efficacy in 37 patients, and Heiskala et al. (37) reported only one success out of 30 patients. Kunnanayaka et al. (38) compared the combination of pyridoxine–prednisone vs. prednisolone alone in a population of 62 infants and did not find any significant difference. In the study published by Kaushik et al. (39), 135 out of 144 patients with epileptic spasms received pyridoxine, with partial response (>50% improvement) in 12 cases (8.9%) and complete spasm cessation in only 1 patient (0.7%). Thus, high-dose Vitamin B6 might have some antiepileptic action in West syndrome, but the level of evidence is very low.

In conclusion, data from the literature and data analysis of this cohort of patients suggest that *de novo* West syndrome is not a typical feature of PDE or PNPO dependency. Spasms are among the types of seizures presented by patients with PDE but, unlike *de novo* West syndrome, these patients usually present other types of seizures, have an earlier epilepsy onset (predominantly in their first month of life), and do not present hypsarrhythmia but other types of EEG abnormalities, like the SB pattern. We think that it is not legitimate to propose a systematic pyridoxine or PLP trial in classical *de novo* West syndrome as it is likely to delay the use of other therapeutic strategies, such as hormonal therapy and vigabatrin, which could have a strong negative impact on the long-term outcome (19, 20). This is in line with the recommendations of the American Academy of Neurology and of the Practice Committee of the Child Neurology Society for treatment of West syndrome (40). On the other hand, vitamin trials and metabolic screening should be considered when the epileptic spasms have an onset before the age of 2 months, are associated with a SB aspect on EEG, are preceded by other types of seizures, or do not respond to first-line treatments.

## Data Availability Statement

The original contributions presented in the study are included in the article/supplementary material, further inquiries can be directed to the corresponding author/s.

## Ethics Statement

Ethical review and approval was not required for the study on human participants in accordance with the local legislation and institutional requirements. Written informed consent from the participants' legal guardian/next of kin was not required to participate in this study in accordance with the national legislation and the institutional requirements.

## Author Contributions

MG and MB: study design, data collection, data analysis, data interpretation, drafting and revising the manuscript. JL, KM, NV, MS, HM, M-AB, IC, MC, DD, MK, M-DL, AR, and RN: data collection, data analysis, data interpretation, drafting and revising the manuscript. PV: study design, data analysis, data interpretation, drafting and revising the manuscript. All authors contributed to the article and approved the submitted version.

## Conflict of Interest

The authors declare that the research was conducted in the absence of any commercial or financial relationships that could be construed as a potential conflict of interest.
